# Inter-Brain Synchronization During Sandplay Therapy: Individual Analyses

**DOI:** 10.3389/fpsyg.2021.723211

**Published:** 2021-11-23

**Authors:** Michiko Akimoto, Takuma Tanaka, Junko Ito, Yasutaka Kubota, Akitoshi Seiyama

**Affiliations:** ^1^Faculty of Human Sciences, Toyo Eiwa University, Yokohama, Japan; ^2^Faculty of Data Science, Shiga University, Hikone, Japan; ^3^Faculty of Health Sciences, Kyorin University, Tokyo, Japan; ^4^Health and Medical Services Center, Shiga University, Hikone, Japan; ^5^Human Health Sciences, Graduate School of Medicine, Kyoto University, Kyoto, Japan

**Keywords:** social interaction, sandplay therapy, NIRS, hyperscanning, nonverbal psychotherapy, synchronization

## Abstract

Interactions between the client (Cl) and therapist (Th) evolve therapeutic relationships in psychotherapy. An interpersonal link or therapeutic space is implicitly developed, wherein certain important elements are expressed and shared. However, neural basis of psychotherapy, especially of non-verbal modalities, have scarcely been explored. Therefore, we examined the neural backgrounds of such therapeutic alliances during sandplay, a powerful art/play therapy technique. Real-time and simultaneous measurement of hemodynamics was conducted in the prefrontal cortex (PFC) of Cl-Th pairs participating in sandplay and subsequent interview sessions through multichannel near-infrared spectroscopy. As sandplay is highly individualized, and no two sessions and products (sandtrays) are the same, we expected variation in interactive patterns in the Cl–Th pairs. Nevertheless, we observed a statistically significant correlation between the spatio-temporal patterns in signals produced by the homologous regions of the brains. During the sandplay condition, significant correlations were obtained in the lateral PFC and frontopolar (FP) regions in the real Cl-Th pairs. Furthermore, a significant correlation was observed in the FP region for the interview condition. The correlations found in our study were explained as a “remote” synchronization (i.e., unconnected peripheral oscillators synchronizing through a hub maintaining free desynchronized dynamics) between two subjects in a pair, possibly representing the neural foundation of empathy, which arises commonly in sandplay therapy (ST).

## Introduction

Psychotherapy is a unique form of communication among all types of social interactions between two individuals: a client (Cl) and a therapist (Th). According to Fuchs (2004, p. 482). successful psychotherapy is denoted by the mutual creation of meaning, which is not a “state in the head,” but rather arises from a system “between” the Cl and Th. The processes of psychotherapy depend on intentional/conscious endeavors of mutual understanding and automatic/unconscious inferential processing of mental states. An interpersonal link or therapeutic space is implicitly developed, wherein certain important elements are expressed and shared. Furthermore, this relationship is based on a therapeutic alliance, which is considered essential for positive change (Goldfried, 2013; Tschacher et al., 2015). In this study, we investigated therapeutic alliances and their neural backgrounds in sandplay therapy (ST), a unique nonverbal therapeutic modality.

Pioneered by Lowenfeld (1950), ST was further developed as a useful and powerful art/play therapeutic technique by Kalff (2020). As this type of therapy is non-verbal, it can foster strong therapeutic relationships with children and Cls with brain damage, who experience difficulty in expressing themselves verbally (Akimoto, 1995). The uniqueness of sandplay therapy, different from other non-verbal art therapies, lies in its primary material: sand. By touching the sand, the client’s playful imagination is stimulated, thus inducing dynamic process of sand “play” which evolves with time. The sand also connects, through both tactile and visual senses, the body and mind and brings about a powerful healing force. The Th who watches is also exposed to the power of the sand through sight, and the two share a creative space. ST can effectively facilitate transformation and growth in Cls, who are supposed to create a sandtray in a sandbox by molding the sand and using miniature figures to express their inner state. During this procedure, the Th is present to create a “free and protected space” without intervening (Kalff, 2020, p. 16). Afterward, the “sandtray becomes the interactive field between the analysand and analyst, and the sand picture is the visible and tangible form given by the analysand to this special interaction” (Ammann, 1991). The Th’s “empathic understanding and the atmosphere of trust” (Ammann, 1991), which is similar to “mother-child unity” (Kalff, 2020, p. 5), and may even correspond with mother-child synchrony (Feldman, 2012; Leclère et al., 2014), is essential for the transformation of the Cl (Ammann, 1991; Kalff, 1991). Although, the Th seems to be just watching, he or she is actively involved in the Cl’s inner world, and the sandtray created is considered a joint product. However, the healing mechanism of ST remains to be fully elucidated, and neuroscientific clarification is required. Only a few studies have investigated the neural basis of ST. In a recent study examining the intra-brain hemodynamics of a sandplayer during therapy, near-infrared spectrocopy (NIRS) signals from various prefrontal and superior temporal channels were found to be temporarily correlated (Akimoto et al., 2018). Moreover, Foo et al. (2020) assessed the metabolic changes in the bilateral thalami of an individual with generalized anxiety disorder. Using magnetic resonance spectroscopy, the N-acetyl aspartate to creatine (NAA/Cr) ratio (i.e., a measure of neuronal viability) was found to have improved after 18 sessions of ST. Although, these studies may mark important steps toward neuroscientific research on ST, much remains to be addressed in terms of research. Thus, we may be able to understand the effectiveness of ST if we explicate the Cl-Th relationship, which is considered an important therapeutic factor. The invisible bond between Cl and Th may present itself as a connection between the brain; however, no study to date has examined the relationship between Cl and Th in ST using neuroscientific methods.

Emerging research in social neuroscience has clarified that there are some neural substrates of empathy, namely the mirror neuron system (Rizzolatti and Craighero, 2004; Gilbert et al., 2006; Schippers and Keysers, 2011; de Greck et al., 2012; Molenberghs et al., 2012) or mentalizing network researchers (Frith and Frith, 2006; Gilbert et al., 2006). Hyperscanning (Montague et al., 2002), the simultaneous brain scanning of two or more individuals, has enabled the exploration of neural underpinnings concerning dynamic human behavior during interactions among two people or groups. Such analyses include functional MRI (fMRI; Stephens et al., 2010; Schippers and Keysers, 2011; Koike et al., 2016), electroencephalography (EEG; Astolfi et al., 2010; Dumas et al., 2010; Müller et al., 2013; Sänger et al., 2013; Konvalinka et al., 2014), and NIRS studies (Funane et al., 2011; Cui et al., 2012; Dommer et al., 2012; Jiang et al., 2012; Osaka et al., 2015; Baker et al., 2016; Nozawa et al., 2016, 2019; Pan et al., 2017).

Past studies have substantiated the synchronous activities between the brains of subjects participating in cooperative or communicative tasks. For instance, Astolfi et al. (2011) used EEG to investigate cortical synchronization during a cooperative game, observing that the pattern of inter-subject connectivity under the cooperation condition was denser in comparison to defect (i.e., uncooperative) behavior. Using fMRI, Schippers and Keysers (2011) observed the mirroring of the temporal structure of activities in a putative mirror system of two subjects playing charades. Bilek et al. (2015) employed an fMRI two-person approach to identify coupling of the temporoparietal junction (TPJ) in a joint attention task. Several researchers also investigated brain activity during socially meaningful communication with affective components. For instance, Pérez et al. (2017) utilized EEG to measure verbal information interchanges between pairs of subjects about their preferences and opinions on different topics. They demonstrated the existence of brain-to-brain entrainment during the oral narrative, which could be attributed to the shared speech signal or verbal communication itself. Another recent NIRS study by Nozawa et al. (2016) revealed frontopolar (FP) neural synchronization among a group of participants engaging in a cooperative word-chain game that required guessing other members’ thoughts. A NIRS study by Liu et al. (2017) highlighted increased interbrain synchronization when speakers told a story and listeners successfully understood it. For non-verbal tasks, Osaka et al. (2015) found neural synchronization of the left inferior frontal cortex (IFC) for both cooperative singing and humming using NIRS. Vanzella et al. (2019) performed NIRS-based hyperscanning on violin duets, finding different brain activations for the leader and the follower. Specifically, the follower denoted a greater oxy-hemoglobin (oxy-Hb) activation in the temporo-parietal and somatomotor regions during the duo condition than while playing solo, whereas there were no significant differences in the activation of these areas in both duo and solo conditions during the performance of the leader. As for another nonverbal cooperative NIRS study, pairs completing tangrams together showed greater interpersonal neural coordination compared to pairs who completed the same task individually (Fishburn et al., 2018).

These studies have confirmed neural synchronization among two or more people during social interactions. As mentioned above, brain activity has been comprehensively investigated during socially meaningful communication with affective components. However, such profound/empathetic relationship formation, as in psychotherapy, has seldom received attention. Recently, Zhang et al. (2018, p. 104, p. 106) noted that inter-brain synchronization (IBS) was significantly associated with the “bond” (i.e., a component of alliance) between partners in psychological counseling, as opposed to controls. This type of psychotherapy is a verbal exchange, whereby the Cl and Th receive continuous feedback from each other about the effectiveness of their actions. The interaction is more implicit in ST, with a veteran Th perceiving the bond in the sandtray co-created by the Cl and Th. Using music and imagery, Fachner et al. (2019) gauged the brain activity of an experienced Th and Cl with dual-EEG in a real therapy session, discovering a correlation in the time series of frontal asymmetries in parallel with emotional peaks. Nevertheless, neural correlates in the Cl–Th relationship concerning nonverbal therapy have rarely been deliberated. In the context of non-verbal psychotherapy, we used a similar second-person approach (Schilbach et al., 2013) to inspect natural social encounters, wherein emotional engagement and empathic responsiveness are essential.

Therefore, this study aimed to examine the Cl-Th relationship’s neural correlates in simulated ST. Notably, NIRS is non-invasive, easy to set up, and has superior temporal resolution compared to fMRI (Kasai et al., 2015). This optical method makes it possible to examine subjects in a sitting position, while speaking or moving in naturalistic situations, given the robust tolerance to movements (Kasai et al., 2015; Balardin et al., 2017). Hence, studying real-world social interactions is rendered viable, as observed in ongoing ST, wherein Cls rely on their hands to create a sandtray. We hypothesized that spatio-temporally organized neural representation, distinct from verbal interaction, would emerge in parallel with the process of sandplay between Cl and Th pairs.

We tested whether any spatio-temporally significant correlation patterns corresponding to the processes of sandplay might appear through the following three types of comparisons: (1) within-subject (intra-brain) synchronization, (2) between-subjects (inter-brain) synchronization, and to determine whether the synchronization of Cl–Th pairs was indeed induced by being together and performing roles during sandplay sessions, we added (3) pseudo controls constituting all possible combinations of Cl–Th pairs who participated in different sandplay sessions (IBS of unrelated dyads). Correlations between NIRS signals from channels covering cross-hemispheric/homologous regions of these unrelated pairs were also calculated. In this study, the number of subjects was small, and we focused on individual analysis because in sandplay, the relationship between the Cl and Th is not direct but indirect, mediated by play, which greatly varies from Cl to Cl and from pair to pair, and that is why, to date, case studies have been dominant in sandplay research. If synchronization exists, therefore, the pattern will be likely to be different for each Cl–Th combination. We conducted an experiment based on this expectation.

## Materials and Methods

### Participants

Altogether, six volunteer Cls and six volunteer Ths participated in this study. Volunteers were recruited from local universities, including healthy undergraduate or graduate students as Cls, with no history of psychiatric or neurological disorders. The inclusion criteria for volunteer Ths were: (1) either under training in clinical psychology and ST or a practicing clinical psychologist, with (2) no history of psychiatric or neurological disorders. The age of Cls ranged from 19 to 28years (i.e., mean age=21.5years, SD=3.0) and between 23 and 49 years for Ths (i.e., mean age=39.7years, SD=8.2). They formed six (one male-male, one male-female, and four female-female) pairs; however, due to technical problems with NIRS data acquisition, one female-female pair was excluded from the analysis, and the remaining five pairs were analyzed. All participants evaluated in this experimentation were right-handed, as assessed by the Edinburgh Handedness Inventory (Oldfield, 1971).

Written informed consent was obtained from all participants after the experimental procedure was fully explained. The committee on Research Ethics and Interest Conflict of Toyo Eiwa University and the Ethics Committee of Kyoto University approved all aspects of the study protocol, and the methods were performed according to relevant guidelines and regulations.

### Experimental Setting

The experiments were performed in a quiet, shielded room of the Integrated Neuroscience Research Project at the Tokyo Metropolitan Institute of Medical Science. After brief introductions, the Ths instructed Cls how to perform sandplay before setting up the NIRS probes on their heads. The Cl was free to present a scene in the sandbox using miniature objects within 10min, and then answered questions asked by the Th. The participants were seated facing each other either by 90° or 180° (three pairs each) across a long table (74×148×70cm), on which a mini-size sandbox (39×48×6cm) and miniature objects for sandplay were placed. During the NIRS setup, the pairs and experimenters engaged in small talk so that both could relax and become familiar with one another. The Th was allowed to behave as naturally as possible, except that both Cls and Ths were requested not to move their heads to avoid excessive artifacts.

### Sandplay Materials

In total, 66 miniature objects of various categories (e.g., dolls, animals, fish, seashells, coral reefs, flowers, trees, houses, furniture, pianos, ladders, lighthouses, shrines, and bridges) were placed within the sight and reach of the Cl on the right and left sides of the sandbox. Objects that were likely to induce anxiety were excluded to ensure a basic sense of security. However, objects likely to evoke an image of water, such as the sea or a river (e.g., seashells, fish, coral reefs, bridges, and lighthouse), were included.

### NIRS Measurement

We used multichannel NIRS (FOIRE-3000; Shimadzu, Kyoto, Japan) to measure the time courses of concentration changes regarding oxy- and deoxy-Hb during a simulated sandplay session (i.e., 10min at maximum) and post-sandplay interviews (i.e., approximately 5min). Near-infrared light was used at wavelengths of 780, 805, and 830nm, and the time resolution was set to 0.13s.

**Figure 1:**
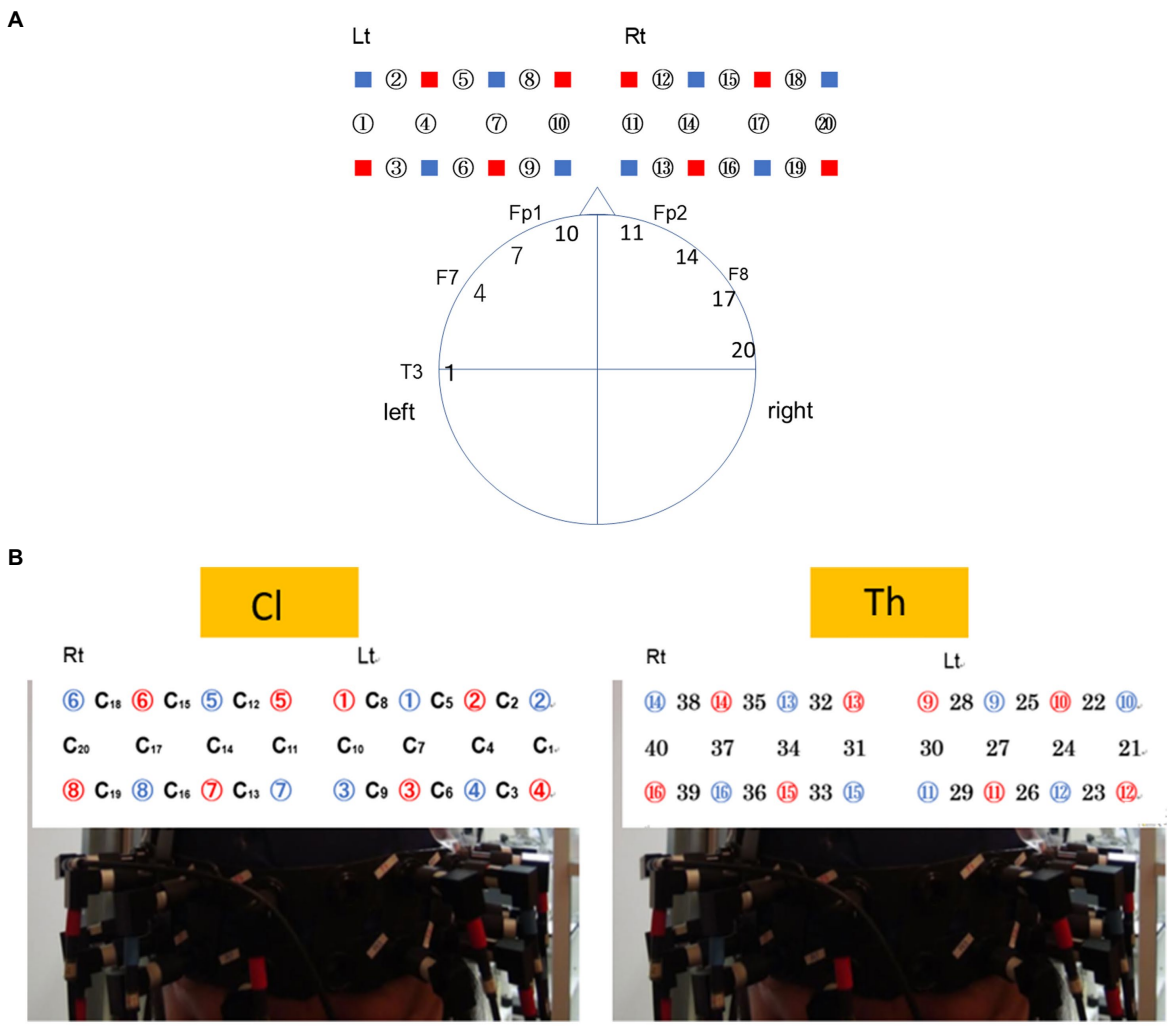
Configuration of measurement channels of the near-infrared spectroscopy (NIRS) signals system based on the 10–20 system. **(A)** Probe placement for the client (Cl). Channels ch9, ch6, and ch3 were located along the line connecting Fp1–F7 of the 10–20 system. The head circumferences of the participants were 55–60cm. The channels ch10/ch7 were placed 3 and 6cm lateral from the central line. Thus, ch10 roughly corresponds to Fp1 (BA10), and ch7 to AF (BA46). **(B)** Probe placement when NIRS is attached to the head of the Cl (left) and therapist (Th; right), respectively. The numbers in red circles indicate emitters, and the ones in blue circles are detectors. The numbers with or not with C indicates channels.

The concentration changes in oxy-Hb and deoxy-Hb were calculated according to the modified Beer-Lambert law for highly scattering media. To simultaneously measure hemodynamics in the Cl–Th pairs, 16 emitters and 16 detectors from one NIRS machine were divided into two halves, and eight emitters and eight detectors were placed into a holder comprising a 2×8 array, which resulted in a total of 20 measurement channels for each subject. Following the international 10–20 system of electrode placement (refer to Figures 1A,B), these channels covered the bilateral prefrontal and superior temporal cortices, with a separation of 3cm. Arrays were attached to swimming caps positioned on each participant’s head so that the prefrontal and superior temporal cortices were covered. The swimming cap was placed low over the forehead and just above the eyebrows of Cls. For both Cls and Ths, the midpoint between detector 7 on the right and detector 3 on the left was 3cm above the nasion, and detectors 7 and 3 were 6cm apart. Channels 1–21 were assigned to Cls, and Channels 22–40 were assigned to Ths.

An initial resting state of 30s served as a baseline. Subsequently, the Ths told Cls to commence the sandplay. The Cls were instructed to turn on an LED lamp to indicate the completion of a sandtray, or the main experimenter stopped the procedure through a microphone. Next, a 5-min semi-structured interview was conducted by the Th so that the Cl’s thoughts and feelings during the sandplay could be verbalized and shared. In the interview, the Th was expected to include the following five questions:

1. What were you thinking during the sandplay? (thoughts).
2. How were you feeling? (feelings).
3. Were there any parts or items that you thought were particularly important or could elaborate on? If so, how did you feel about them? (special memories or complexes).
4. Were there any moments when ideas dawned upon you? (insights or inspiration).
5. Were there any moments when you had some gut feeling or a “This move is just right” feeling? (insights or gut feelings).

The entire session was videotaped by two cameras (Sony HANDYCAM HDR-CX630V) from different angles.

### Data Analysis

From the sandplay task period and the interview session, which lasted approximately 10min, a sampling window of 200s was selected for each session, with relatively stable signals, and without apparent artifacts. To investigate the neuronal background of well-synthesized and emotionally committed interactions between two persons, and to avoid unstable signals often observed in the first part of measurements, we sampled signals from the middle part (200–400s from the start) of the sandplay and interview session.

#### Analysis 1

A band-pass filter was applied to the raw time series of the oxy-Hb signals between 0.01 and 0.1Hz. Oxy-Hb is the most sensitive indicator for changes in regional blood flow (rCBF; Hoshi et al., 2011). We selected the following three connectivities and compared Spearman’s rank-order correlation coefficients to ascertain synchronization between channels during sandplay:

1. Inter-hemispheric synchronization in a subject (intra-brain): connectivity was examined between the inter-hemispheric homologous regions: within-subject condition (10 pairs).
2. Cross-hemispheric synchronization between subjects engaged in the same sandplay (inter-brain): the Cl-Th pair condition (20 pairs).
3. Cross-hemispheric synchronization between all possible combinations of pairs who separately participated in different sandplays (inter-brain): the control (unrelated pairs) conditions (160 pairs).

The numbers of channel pairs were determined as follows: For the intra-brain condition, the selected channel pairs were Cl No. *i*–Cl No. *i* and Th No. *i*–Th No. *i* (*i*=1–5 for Cls and Ths; thus, 10 pairs for each channel pair; e.g., Cl No. 1_L vs. Cl No.1_R and Th No.2_L vs. Th No.2_R). For the inter-brain condition, the selected pairs were Cl No. *i*–Th No. *i*, differentiated depending on whether the Cl–Th channels were from the left (L) or right (R) hemisphere (L–L, L–R, R–L, R–R; *i*=1–5; 20 pairs; e.g., Cl No.1_L vs. Th No.1_L, Cl No.1_L vs. Th No.1_R, Cl No.1_R vs. Th No.1_L, Cl No.1_R vs. Th No.1_R, etc.). For the control condition, the selected pairs were Cl No. *i*–Cl No. *j*, Cl No. *i*–Th No. *j*, and Th No. *i*–Th No. *j* pairs, where *i*≠*j* (*i*, *j*=1–5). Thus, $\left(\begin{array}{c} 5\\ 2 \end{array}\right)=10$ combinations for *i* and *j*, four combinations for Cl and Th, and four combinations for L and R yields 160 pairs (e.g., Cl No.1_L vs. Cl No. 2_L, Cl No.1_L vs. Cl No.2_R, Cl No.1_L vs. Th No.2_L, Cl No.1_L vs. Th No.2_R, etc.; see Figure 1; Supplementary Figure S1).

Notably, the data from the left or right channels encompassing the homologous hemispheric regions were not differentiated in conditions (2) and (3). Examination of the inter-brain, prefrontal cortical synchronization patterns in the rostro-caudal axis and the identification of channels showing prominent inter-brain correlations were the main objectives of this study.

#### Analysis 2

To further investigate the NIRS signal synchronization between the Cl and Th pairs, we calculated the cross-spectrum between signals from the homologous channels of Cls and Ths.

## Results

### Sandplay Picture

**Figure 2:**
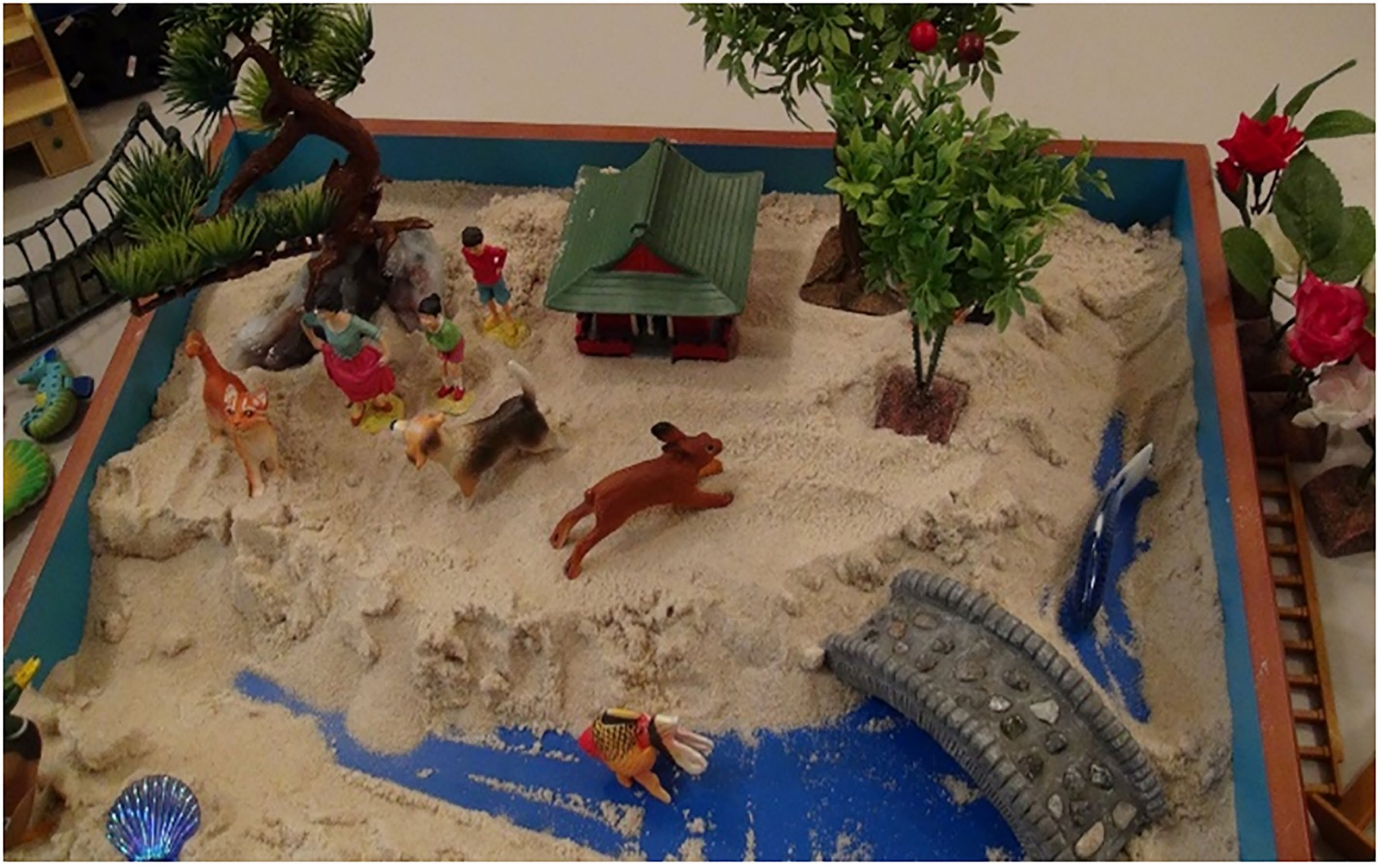
A sample sandplay picture. A sandplay picture made by a female Cl (Pair No. 4). She said that she was totally absorbed in sandplay and said, “I made a river and a pond. The river flows out from the river.” “I wanted to place many animals on the riverbank. I made a happy scene,” “I like it. I’m very satisfied.”

Figure 2 shows a sample sandtray (sandplay picture) made by a Cl (Pair No. 4).

### Results of Correlation Analysis Across Subjects

**Figure 3:**
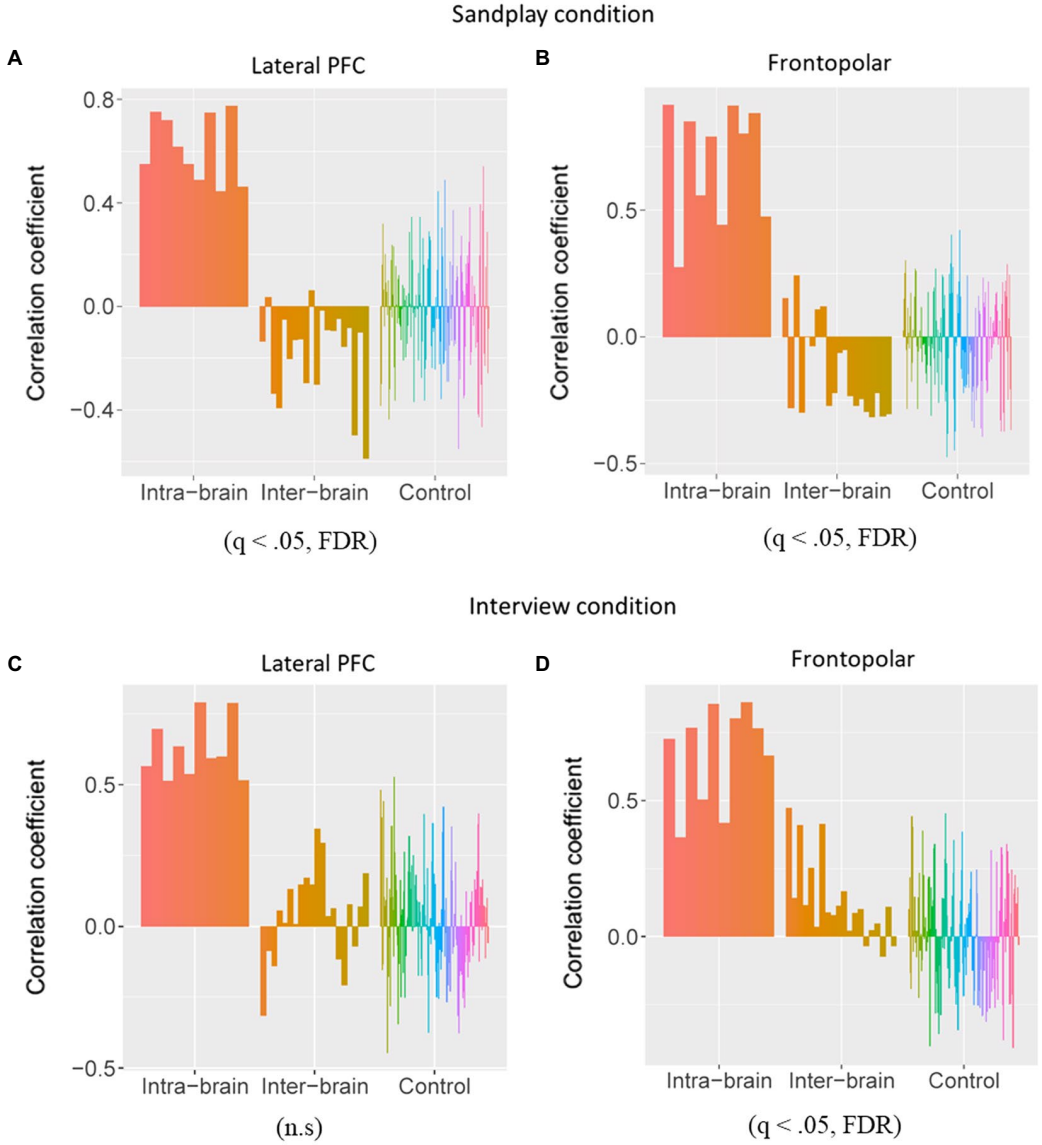
Comparisons of the Spearman rank-order correlation coefficients between the Cl–Th (inter-brain) pairs and the control pairs. The correlation coefficients were calculated within the subjects (intra-brain), between the Cls and Ths who interacted in sandplay (inter-brain), and between control pairs of Cls and Ths who had never actually met (inter-brain). **(A,B)** Under the sandplay condition, significant negative correlations between the pairs were observed in the lateral prefrontal cortex (LPFC) and frontopolar (FP) regions. **(C,D)** During the interview condition, however, a significantly different positive correlation was observed between the pairs in the FP regions, but no significant results were obtained for the LPFC.

**Figure 4:**
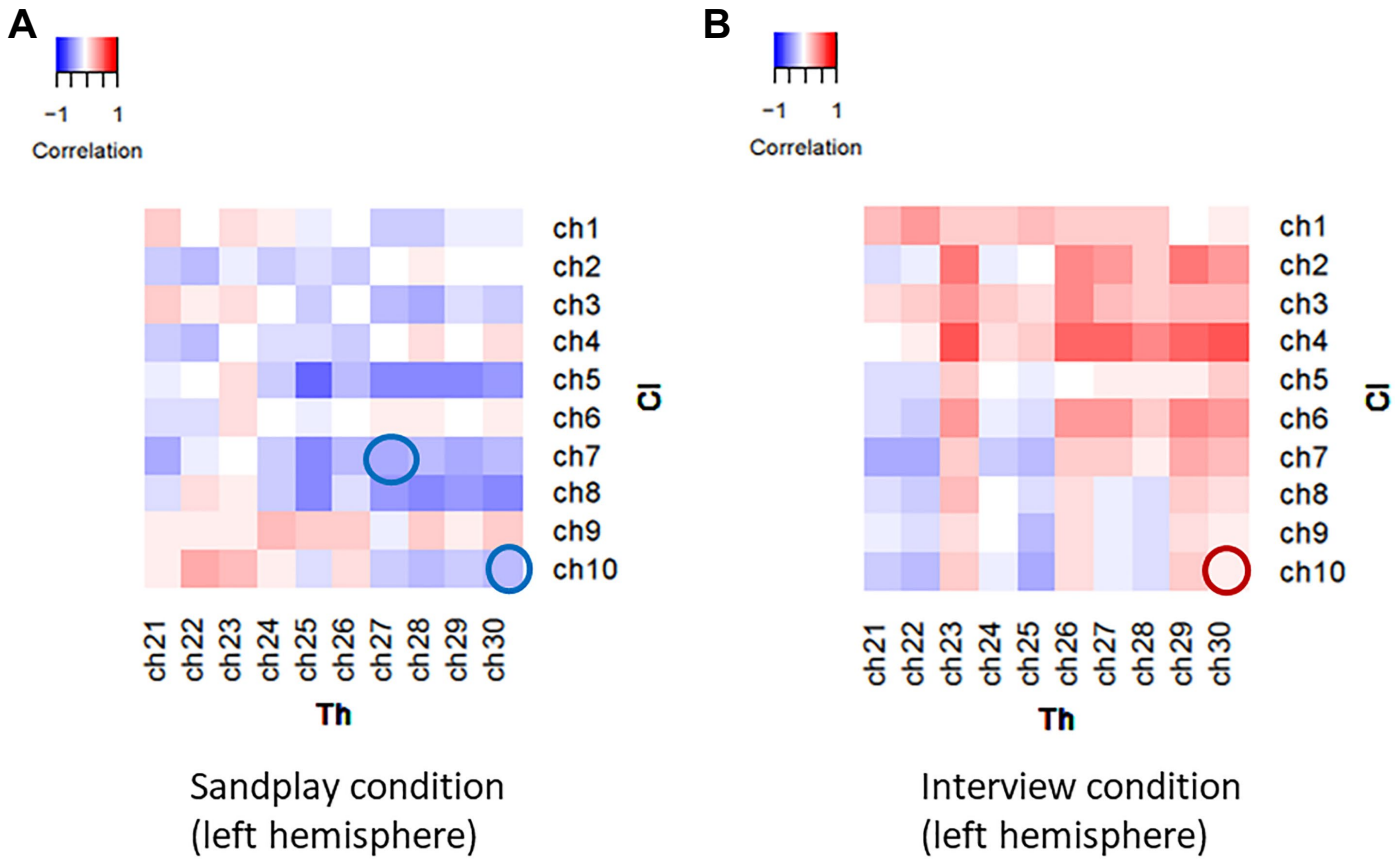
Heatmaps of the correlational values of the NIRS signals between channels in a representative Cl–Th pair (Pair No. 3). Spatio-temporally distinctive correlational patterns were observed during sandplay **(A)** and the interview **(B)**. During sandplay, negative correlations were observed in the FP and lateral PFC channels (blue circles). In contrast, during the interview, a positive correlation was observed in the FP channel (red circle).

Under the three above-mentioned conditions, Spearman’s rank coefficients were used to assess the correlations between the two channels. The Mann−Whitney U test was employed to compare the correlation values between the Cl–Th pairs and control conditions. During sandplay, the difference in the Spearman’s rank-order correlation coefficients between the Cl–Th pairs and the control pairs were statistically significant in the channels covering (1) lateral PFC (LPFC) and (2) FP regions [denoted as channel 7 and 10 in Figures 3A,B, respectively (*q*<0.05, FDR)]. For the LPFC, same-simultaneous pairs are those of channels within a subject (intra-brain), that is, Cl*_i_*-Cl*_i_* (ch7–ch14; *n*=5) and Th*_i_*-Th*_i_* (ch27–ch34; *n*=5) pairs (10 pairs). Simultaneous pairs are those of channels belonging to subjects participating in the same sandplay sessions (inter-brain), that is, Cl*_i_*-Th*_i_* (ch7–ch27, ch7–ch34, ch14–ch27, and ch14–ch34) pairs (*n*=4×5=20). Control pairs are those of corresponding channels found among randomly matched subjects who were previously unacquainted (inter-brain), that is, Cl*_i_*-Cl*_j_*, Cl*_i_*-Th*_j_*, and Th*_i_*-Th*_j_* pairs, where *i*≠*j* (*n*=160). For the FP regions, same-simultaneous pairs are those of channels within a subject (intra-brain): Cl*_i_*-Cl*_i_* (ch10–ch11) and Th*_i_*-Th*_i_* (ch30–ch31) pairs (10 pairs). Simultaneous pairs are those of channels pertaining to subjects involved in the same sandplay sessions (inter-brain): Cl*_i_*-Th*_i_* (ch10–ch30, ch11–ch31, ch10–ch31, and ch11–ch30) pairs (4×5=20 pairs; Figures 3A,B). Control pairs are those of corresponding channels related to subjects who never became partners (inter-brain): Cl*_i_*-Cl*_j_*, Cl*_i_*-Th*_j_*, and Th*_i_*-Th*_j_* pairs, where *i*≠*j* (160 pairs). Contrariwise, significant differences were observed only in channels covering the FP region during the interview (denoted as channel 10; *q*<0.05, FDR). Generally, synchronization between signals from the LPFC and FP channels showed a negative correlation under the sandplay condition (Positive correlation was found only in Pair No. 1 in the FP), whereas in the interview condition, predominantly positive correlations were observed in the FP channels and no correlations were evident in the LPFC channels (Figures 3A–D). The Spearman’s rank-order correlation coefficients of the channels in the left hemispheres of simultaneous pairs are presented as heatmaps (Pair No. 3 in Figures 4A,B; Pair No. 1, 2, 4, and 5 in Supplementary Figure S2).

### Results of Cross-Spectrum Between Channels

**Figure 5:**
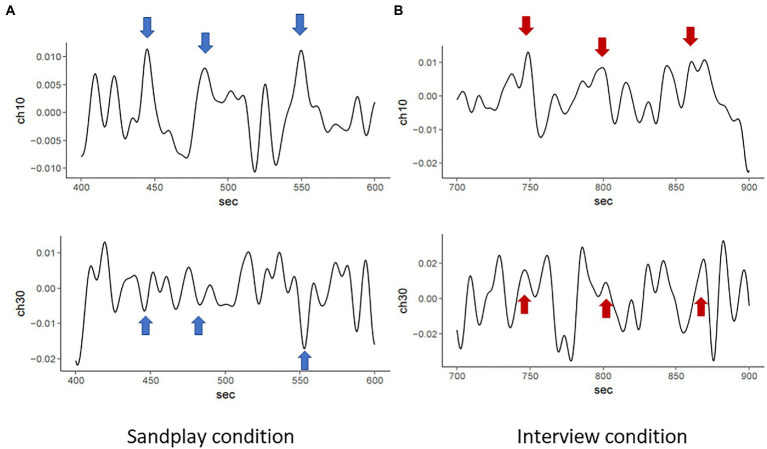
Raw NIRS signals from FP channels of the same pair (Pair No. 3). During sandplay **(A)**, NIRS signals indicating hemoglobin concentration changes of the Cl (above) and the Th (below) showed mirroring patterns (blue arrows), possibly reflecting the Cl–Th interaction during sandplay. These patterns yielded a relatively slow and negative correlation between the Cl and Th. In contrast, during the interview **(B)**, the two channels showed relatively slow and positive correlation (red arrows).

**Figure 6:**
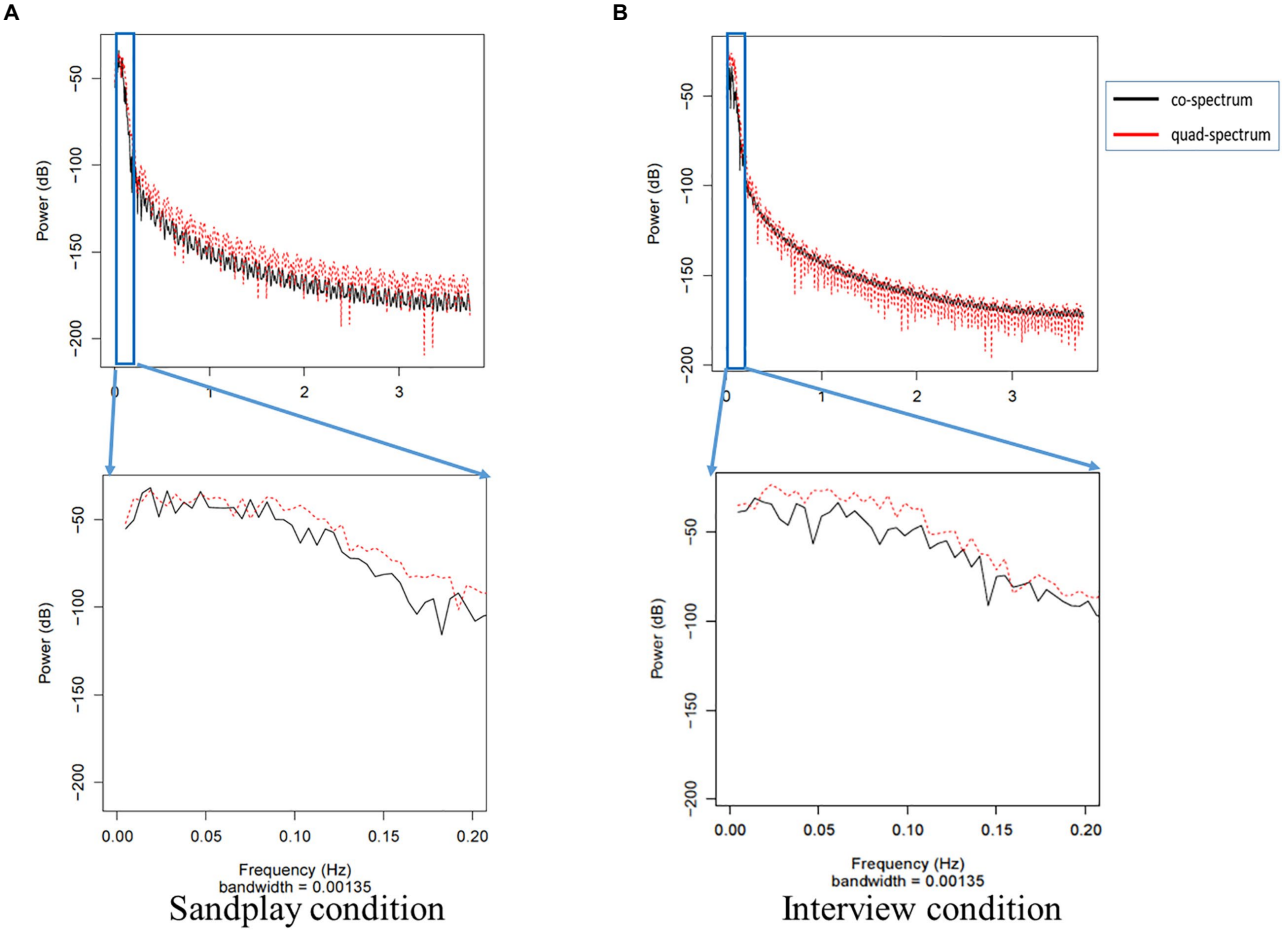
Cross-spectrum of FP signals from the same pair (Pair No. 3) during sandplay **(A)** and the interview **(B)**. In the top power graph, the gray box indicates the power spectrum of slow oscillation. Note that the frequency oscillations of the low-pass and high-pass filters are 0.1 and 0.01Hz, respectively. The black line represents the co-spectrum, and the red line represents the quad-spectrum. These data were expanded in the lower power graph, which shows that the distribution of the power approximately varied from 0.02 to 0.05Hz.

We estimated the cross-spectrum between signals from the LPFC and FP channels described above (denoted as ch7 and ch10). We discovered low-frequency oscillations (i.e., 0.01–0.08Hz). The raw data of changes in hemoglobin concentration, together with cross-spectral densities of one Cl-Th pair (Pair No. 3), are expressed in Figures 5, 6. Additionally, cross-correlograms (see Supplementary Figure S3) emphasize that NIRS signals of the Cl and Th were negatively correlated in the FP channels during sandplay, with a lag of approximately 2s.

## Discussion

The spontaneous synchronization of frontal cortical tissue oxygenation between dyads participating in sandplay was revealed in this study. The synchronous activities, with relatively slow periodicity, signified a spatially localized distribution within the prefrontal area, unlikely elicited by external stimuli perception, motor command, or physiological confounds. Still, this distribution may represent functionally significant oscillatory patterns emerging in the networks between two “connected” brains.

The NIRS techniques allowed real-time measurement of ongoing cortical hemodynamics in a naturalistic condition closely resembling a clinical setting. During the sandplay condition, the Cl engaged with the sandtray without any interventions, and the Th followed that heuristic processes intensively. Depending on the condition, cortical areas showing distinct correlations were distributed in time-varying patterns. The channels covering the LPFC and those covering the most rostral parts (i.e., FP channels) specified distinct negative synchronizations under the sandplay condition. Conversely, only the NIRS channels in the FP region showed a prominent positive correlation during the interview condition, when the Cl and Th were recalling and reflecting on the therapeutic procedures.

Recent diffusion tensor imaging studies on humans have examined the anatomical connection between the FP and LPFC regions (Catani et al., 2012; Moayedi et al., 2015). Functional studies also reported that the lateral FP cluster is connected to the executive control network (Seeley et al., 2007; Weissman-Fogel et al., 2010), which is more active in externally rather than internally focused cognition. These findings can be regarded as an extension of the results mentioned above from a single brain to network activities across two brains. Interestingly, anatomical FP-LPFC connections and functional connectivity are absent in nonhuman primates (Neubert et al., 2014; Saleem et al., 2014). Hence, the observed synchronization might reflect neurocognitive processes unique to humans in terms of neuroanatomy and the social network observed in the therapeutic relationship. Furthermore, the positive synchronous patterns were localized to the FP channels during the interview condition (see Figure 3D). This outcome is consistent with previous hyperscanning studies concerning verbal social interactions (Nozawa et al., 2016). According to Ellamil et al. (2012), activation of the rostrolateral PFC may indicate the involvement of meta-cognitive evaluative processing, including judgments regarding the pursuit of an initial creative idea or goal and the appropriateness of the final creative product. Correspondingly, our results may reflect such internally focused, meta-cognitive reflective processes that evolved from interactions between the two individuals. These findings were somewhat consistent with the hierarchical organization of the PFC system in the rostro-caudal and medial-lateral axes (O’Reilly, 2010).

The results of FP synchronization during the interview were largely in line with those of previous studies (Suda et al., 2010; Nozawa et al., 2016; Liu et al., 2017). However, we also found synchronization in the FP during the naturalistic condition involving the creation of a sand scene. The Cls and Ths did not interact with each other in active and visible ways, and the Ths mostly sat still and observed the process (note: one Th, who kept her eyes closed during sandplay, stated that she was concentrating on listening to the sounds made by the Cl while constructing a sand scene). Nevertheless, interbrain synchronization emerged spontaneously, although, the correlation coefficients were predominantly negative in this condition.

Synchronization between discrete neural circuits of the two spatiotemporally connected brains, having no direct communication, may seem counter-intuitive or even mysterious at first sight, as in the case of pendulum synchronization observed by Huygens (Ramirez et al., 2016). Under the sandplay condition, the findings imply that the synchronization of oscillators is neither directly led by apparent internal/external oscillations, such as paced motor commands or rhythm perception, nor by direct structural or anatomical connections.

Raz et al. (2016) examined the dynamic interactions between specific domain-general networks across time as participants experienced various emotions (e.g., “sadness,” “happiness”), while viewing films (i.e., shared experiences). They demonstrated that the functional connectivity between the networks co-varied with the intensity of emotional experiences that were not unique to a specific emotional category. The present task procedure did not necessarily elicit typical emotional reactions, such as sadness or happiness. Nevertheless, projecting a subjective inner world onto a sand scene (e.g., digging a river, planting trees, or placing a small figurine on the riverbank) almost always represents a deep and personal (i.e., not categorical) emotional experience not only for the Cl but also for the Th who witnesses it. The therapeutic relationship in ST has at times been called “co-transference” (Bradway and McCoard, 1997, p. 34), or “the therapeutic feeling relationship between therapist and client, containing experiential states and feelings that go back and forth simultaneously on conscious and unconscious levels” (Cunningham, 2013, p. 85). Thus, we hypothesized that the observed synchronization between the cortical networks of the brains of both the Cl and the Th was made possible through the sharing of conscious and unconscious experiences regarding sandplay in the therapeutic space, without any forced timings. Indeed, the shared sand picture itself can act as an outwardly expressed symbol of one’s inner state (i.e., a joint product of co-transference), facilitating deep levels of communication between the Cl and Th. To our knowledge, this evidence is the first to signify “remote” synchronization (Vlasov and Bifone, 2017), which is the synchronization of unconnected peripheral nodes through a hub maintaining desynchronized dynamics between two brains, each exploring deep/inner worlds, mediated by the process of creating a sand scene. This study is also the first to integrate a neural basis of empathy within the context of ST.

Previous researchers have shown that independent oscillators can be synchronized using common input (Pikovsky et al., 2001; Teramae and Tanaka, 2004). In this study, the specific pattern of FP-lateral PFC synchronization during the sandplay condition was observed, as Ths were “mentalizing” or “actively observing” the Cls’ action. A possible interpretation of the results is that the perception of sandplay might act as a “common input,” inducing phase synchronization in the oscillators on both sides of the brain.

Typically, the slow oscillatory activities (0.02~0.05Hz) were reported in remote networks within one brain (i.e., intrinsic functional synchronization; Chang and Glover, 2010; Zuo et al., 2010; Baria et al., 2011; Xue et al., 2014; Goelman et al., 2017). For example, Baria et al. (2011) systematically studied whole-brain oscillatory activity for the four bandwidths of frequencies available with fMRI and observed a band-specific spatial structure. Specifically, the largest power in the lowest frequency (LF) band (0.01~0.05Hz) is situated in the medial PFC and the medial and lateral posterior parietal cortices, which together yield the default mode network (DMN). In addition, robust band-dependent power changes due to the visual-motor task were observed; the LF band power showed regional decreases, while much higher frequency bands showed regional increases. These results are largely in line with the present findings and support that connectivity in slow bands may have functional significance. However, it is unclear how synchronicity on the timescale is related to changes in the conscious and unconscious experiences of the participants. Therefore, this aspect needs to be scrutinized in future studies. The functional significance of the negative correlation, which was observed in a previous fMRI study (Goelman et al., 2017), also remained unclear. In this research, a close examination of the hemodynamic fluctuations in the task condition revealed reverse correlations with lags of approximately a few seconds, which are likely to have occurred in response to external stimuli due to (1) time lags between the Cl’s action and the Th’s perception and (2) corresponding changes in brain states reflecting strategic processes for constructing (Cls) and interpreting (Ths) sand scenes.

Due to a lack of verbal explanation, it may have taken Ths some time to perceive what was happening in the sandplay in our case, and meanings may have emerged slowly. ST is often administered as a component of play therapy; thus, it is not always followed by a verbal interaction (e.g., interview). A verbal interaction might likely lead to further clarification and empathic understanding of the content (Badenoch, 2008). In the future, a more powerful time-series analysis that combines NIRS signals with detailed descriptive data concerning subjective experience is required to comprehend how this procedure is manifested as brain coupling.

Several limitations of the present study should be noted. First, the sample size was small; however, the correlations were compared between 20 paired Cl and Th channel sets and 160 random combinations of Cls and Ths who were previously unacquainted. Second, the behavior of the participants was not uniform. The difference in the observing behavior of Ths, especially whether they kept their eyes open or closed, may have affected their understanding of the Cls’ sandplay, although, there is evidence of a human auditory mirror system, which is related to empathy (Gazzola et al., 2006). However, strict control of Cl behavior is likely to undermine the ecological validity of our study. It is recommended that future studies should address the differences in gender as well as therapists’ expertise (years of therapy experience) into consideration. Nevertheless, it is noteworthy that, despite the differences in gender and years of experience in our study, significant correlations were found between the Cl and Th in all pairs. For sandplay, in particular, both externally-oriented (LPFC) and internally-oriented (FP) brain regions (Gilbert et al., 2006; Nozawa et al., 2019) were almost inversely correlated, suggesting that there may be a common “therapist attitude” or “client-therapist relationship” in sandplay, where the scene is co-created with internal feedback and examination by the Cl and Th, and in each case, the Cl–Th bond may be mediated by the common “hub,” which is sandplay.

Third, although, the time resolution is relatively high, the spatial resolution of NIRS is far lower than that of MRI. Thus, precise anatomical information of the cortical regions showing prominent correlation should be obtained in future studies. Fourth, although, we used a band-pass filter, possible systemic effects on cortical hemodynamics cannot be excluded since NIRS measures hemoglobin concentration changes not only in the cerebral cortices but also in extracerebral tissues, such as skin and cerebrospinal fluid. Nevertheless, the present patterns of synchronization were detected in relatively confined regions, and blood pressure or respiration-related fluctuations, if present, should be observed in more spatially widespread cortical regions.

In summary, neural synchronization was observed in the PFC and FP regions among pairs of Cls and Ths engaged in sandplay and post-sandplay interviews. The oscillatory patterns of the two brains were different under these conditions; a negative correlation was found between the partners during the nonverbal condition of sandplay and a positive correlation was observed in verbal communication. As sandplay is a form of psychotherapy accompanied by unique nonverbal unconscious interactions, our preliminary study adds to the growing body of research in terms of real-world social interaction, providing a new possibility to explore the complex and unique Th-Cl relationship, which is a crucial factor for successful psychotherapy.

## Data Availability Statement

The datasets presented in this study can be found in online repositories. The names of the repository/repositories and accession number(s) can be found at: https://datadryad.org/stash/share/tXBk2uHeVhWPSQyn8wi3KpugXsV5Q_XoSShBSXPlwVw.

## Ethics Statement

The studies involving human participants were reviewed and approved by The committee on Research Ethics and Interest Conflict of Toyo Eiwa University and The Ethics Committee of Kyoto University. The patients/participants provided their written informed consent to participate in this study.

## Author Contributions

MA and JI conceived and designed the experiment and acquired the data. MA, TT, JI, YK, and AS processed and analyzed the data. MA, YK, and TT interpreted the results. MA and YK drafted the manuscript, which was revised by all authors. All authors contributed to the article and approved the submitted version.

## Funding

This work was supported by the Japan Society for the Promotion of Science (JSPS) KAKENHI (Grants-in-Aid for Scientific Research), grant no. 25380953.

## Conflict of Interest

The authors declare that the research was conducted in the absence of any commercial or financial relationships that could be construed as a potential conflict of interest.

## Publisher’s Note

All claims expressed in this article are solely those of the authors and do not necessarily represent those of their affiliated organizations, or those of the publisher, the editors and the reviewers. Any product that may be evaluated in this article, or claim that may be made by its manufacturer, is not guaranteed or endorsed by the publisher.
