# Supplementary material for: Inter-Brain Synchronization During Sandplay Therapy: Individual Analyses
Source: Front Psychol. 2021 Nov 23;12:723211. doi: 10.3389/fpsyg.2021.723211 (PMC8650609; doi:10.3389/fpsyg.2021.723211)
Supplement: Supplementary file 1 [file Data_Sheet_1.pdf]

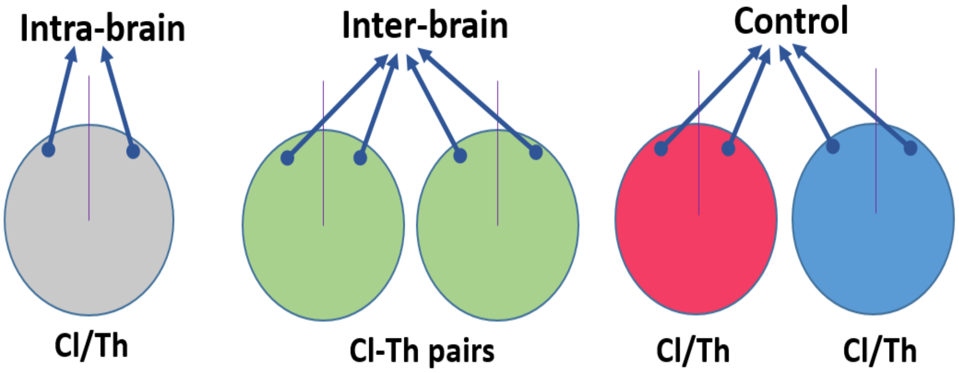

Figure S1 Design for comparison.

The correlation coefficients were calculated within the subjects (intra-brain), between the Cls and Ths who interacted in sandplay (inter-brain), and between unrelated pairs of Cls and Ths who never met (control).

A

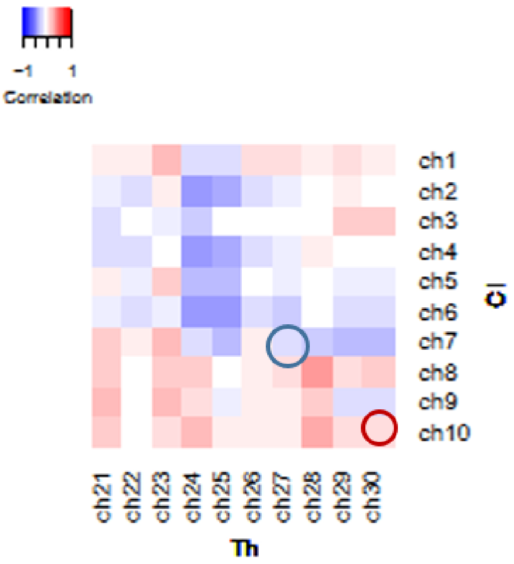

Sandplay condition
(left hemisphere)

B

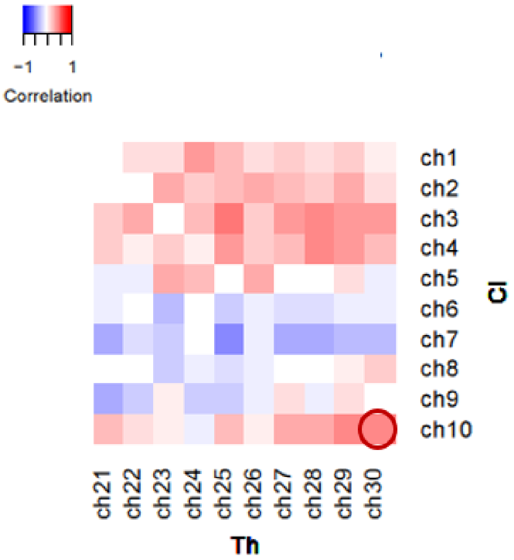

Interview condition
(left hemisphere)

Pair No. 1

C

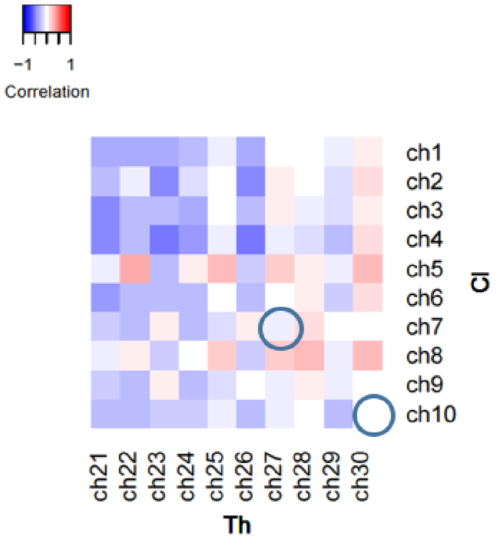

Sandplay condition
(left hemisphere)c

D

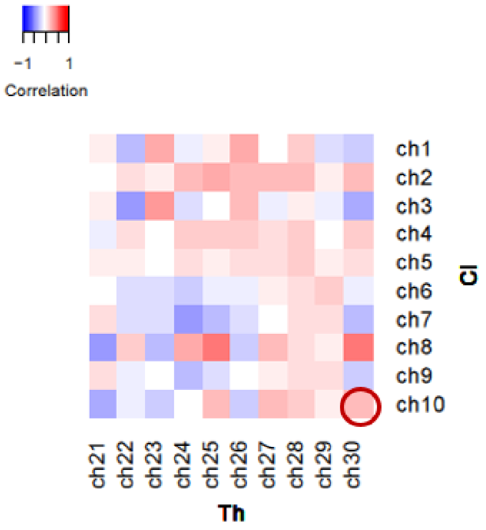

Interview condition
(left hemisphere)

Pair No. 2

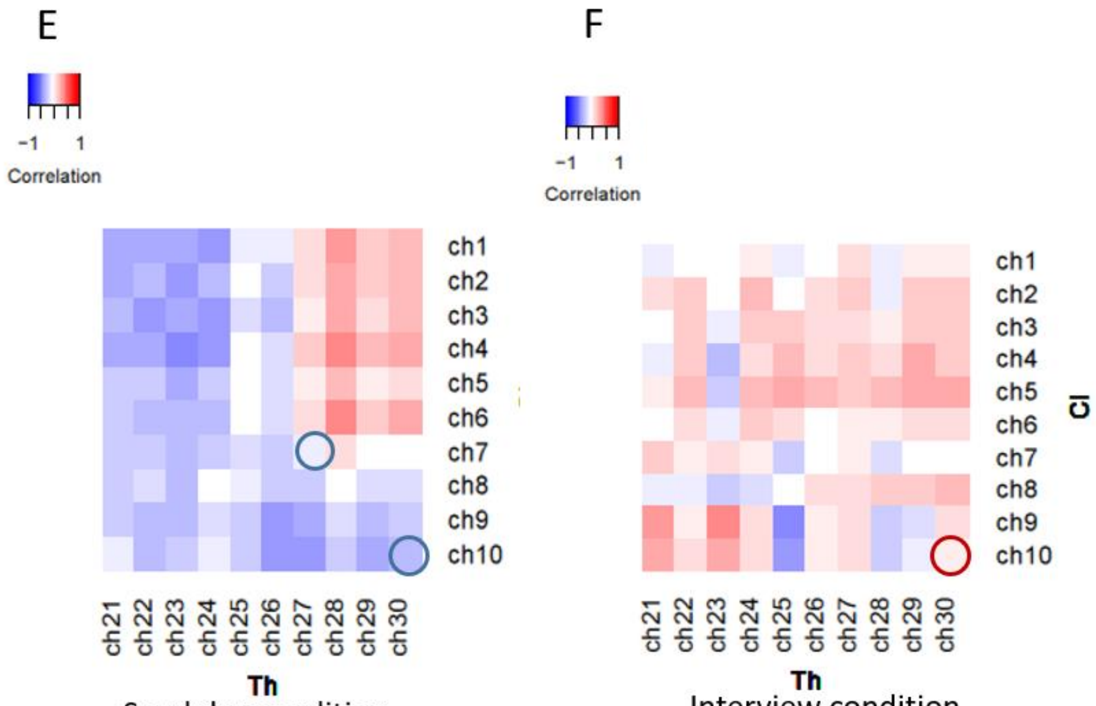

Sandplay condition (left hemisphere)

Interview condition (left hemisphere)

Pair No. 4

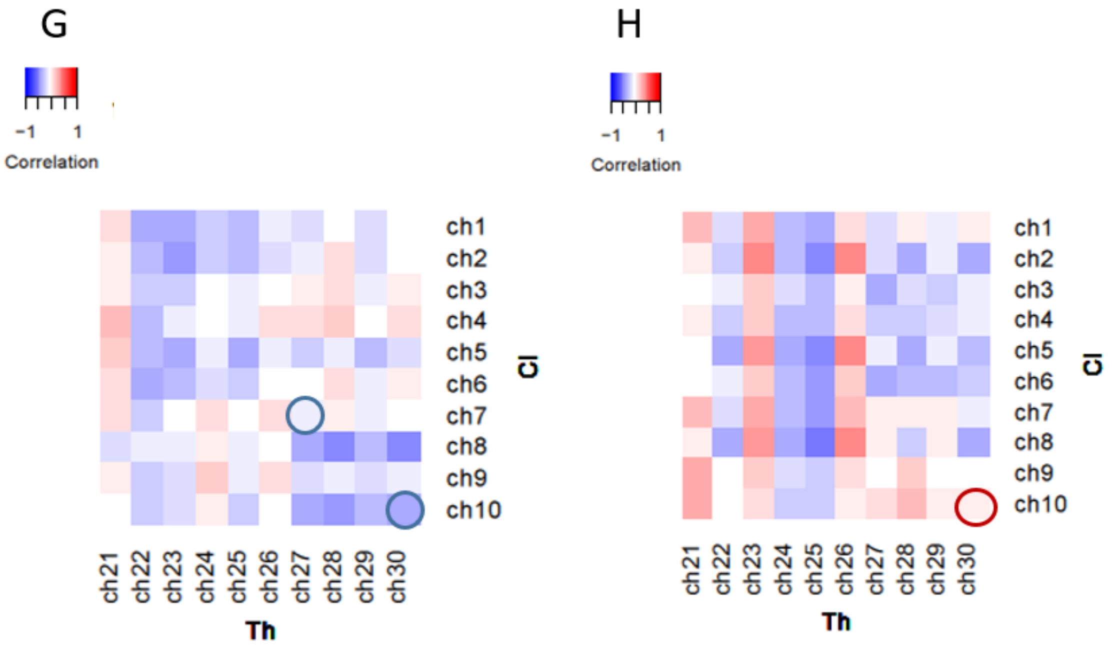

Sandplay condition (left hemisphere)

Interview condition (left hemisphere)

Pair No. 5

Figure S2 Heatmaps of correlational values of the FP signals (left hemisphere) between Cl-Th pairs (Pair 1, 2, 4, & 5).

Spatio-temporally distinctive correlational patterns were observed during sandplay (Figure S2A, S2C, S2E, & S2G) and the interview (Figure S2B, S2D, S2F, & S2H). During sandplay, negative correlations were observed in all the lateral PFC channels (blue circles) in Pairs No. 2-5. Only in Pair No. 1, positive correlation was obtained in the PFC. In contrast, during interview, a positive correlation was observed in the FP channel (red circles) in all pairs.

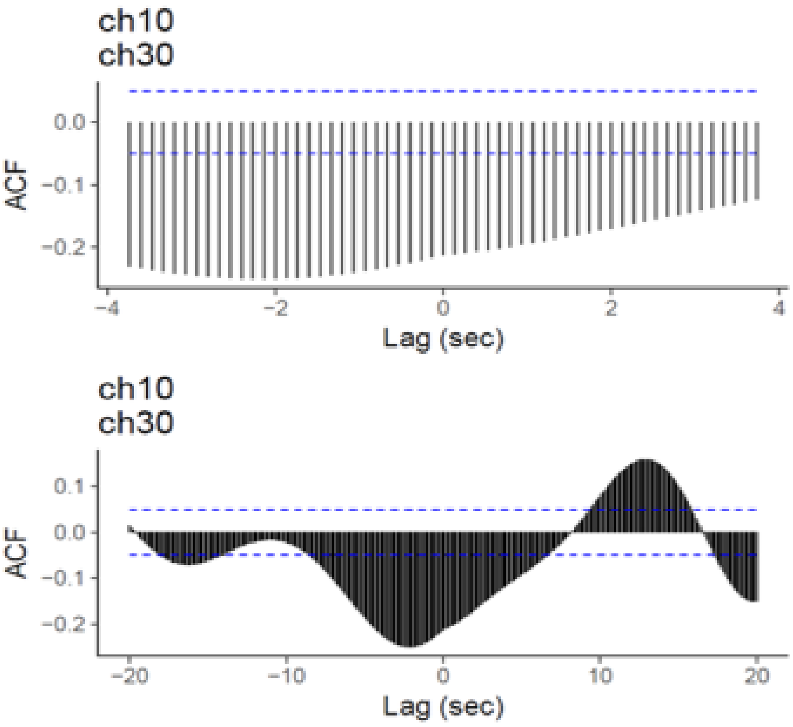

Figure S3 The crosscorrelograms of Pair No.3 during sandplay. The above is an enlarged figure of the crosscorrelogram below. The maximum lag for the above cross-correlogram is 4 seconds and that for the below is 20 seconds. As is shown in Figure 3 in the main text, the NIRS signals of the Cls and Ths were negatively correlated during sandplay, with the Cl lagging behind for approximately 2 seconds.
